# Translational Research in FLASH Radiotherapy—From Radiobiological Mechanisms to In Vivo Results

**DOI:** 10.3390/biomedicines9020181

**Published:** 2021-02-11

**Authors:** Loredana G. Marcu, Eva Bezak, Dylan D. Peukert, Puthenparampil Wilson

**Affiliations:** 1 Faculty of Informatics & Science, Department of Physics, University of Oradea, 410087 Oradea, Romania; 2 Cancer Research Institute and School of Health Sciences, University of South Australia, Adelaide, SA 5001, Australia; Eva.Bezak@unisa.edu.au; 3 School of Physical Sciences, Department of Physics, University of Adelaide, North Terrace, Adelaide, SA 5005, Australia; 4 School of Civil, Environmental & Mining Engineering, University of Adelaide, North Terrace, Adelaide, SA 5005, Australia; dylan.peukert@mymail.unisa.edu.au; 5 STEM, University of South Australia, Adelaide, SA 5001, Australia; Puthenparampil.Wilson@unisa.edu.au; 6 Department of Radiation Oncology, Royal Adelaide Hospital, Adelaide, SA 5000, Australia

**Keywords:** ultra-high dose rate, normal tissue sparing, FLASH-radiotherapy, FLASH-radiobiology, therapeutic window

## Abstract

FLASH radiotherapy, or the administration of ultra-high dose rate radiotherapy, is a new radiation delivery method that aims to widen the therapeutic window in radiotherapy. Thus far, most in vitro and in vivo results show a real potential of FLASH to offer superior normal tissue sparing compared to conventionally delivered radiation. While there are several postulations behind the differential behaviour among normal and cancer cells under FLASH, the full spectra of radiobiological mechanisms are yet to be clarified. Currently the number of devices delivering FLASH dose rate is few and is mainly limited to experimental and modified linear accelerators. Nevertheless, FLASH research is increasing with new developments in all the main areas: radiobiology, technology and clinical research. This paper presents the current status of FLASH radiotherapy with the aforementioned aspects in mind, but also to highlight the existing challenges and future prospects to overcome them.

## 1. Introduction

The aim of radiotherapy is to deliver a tumoricidal dose to the neoplasm while keeping normal tissue toxicity to a minimum. Over the last few decades, radiotherapy has improved via novel technologies, such as image-guided radiation therapy (IGRT) and intensity-modulated radiation therapy (IMRT) and through the clinical implementation of particulate radiation with superior physical and radiobiological properties, as compared to the more-established photons and electrons [1,2]. Regardless of these advances, the research community continuously strives to improve the existing treatments by trialling new ways of increasing the therapeutic ratio. One such example is FLASH radiotherapy, where the evidence so far points towards meeting this goal.

FLASH radiotherapy is a non-conventional technique that delivers dose rates ≥ 40 Gy/s for a single radiation dose [3]. While the biological mechanisms behind FLASH are not fully elucidated, the scientific rationale behind the administration of ultra-high dose rates is the enhancement of the therapeutic window in radiation therapy through a better normal tissue sparing and similar, or an increased, tumour control, as compared to conventional therapies [4].

The aim of this paper is to review the current status of FLASH radiotherapy. The radiobiology of FLASH therapy, the delivery techniques to produce the ultra-high dose rate radiation and the existing pre-clinical and clinical evidence on FLASH radiotherapy are examined. While there are still several challenges regarding both radiobiological and technical aspects, the results of in vitro and in vivo studies are so far reassuring and warrant further investigation.

## 2. Radiobiological Rationale

To date, the radiobiology of FLASH radiation therapy is not fully understood. In most references, this is explained by: (a) oxygen depletion effect, (b) inflammatory processes, (c) redox biology, and (d) differential effect/reaction of normal vs. tumour tissues [4,5,6,7].

Oxygen depletion is considered to have radio-protective effect on normal tissues. Once the oxygen levels have been depleted sufficiently by the initial boost of radiation, the subsequent irradiation of normal tissues occurs in hypoxic conditions, and therefore in a radioresistant state. Additionally, when using high doses and ultra-high dose rates, reoxygenation cannot occur. This may, in effect, separate the window between tumour control probability (TCP) and normal tissue complication probability (NTCP) curves [4].

This is possibly an overly simplistic explanation, and doubts persist as to whether the dose rates used clinically in FLASH radiotherapy are sufficient to significantly affect radiolysis yields. Considering the many biological processes occurring at the subcellular level during irradiation, other processes may be responsible for the clinical effects observed, including chromatin remodelling or inflammatory/anti-inflammatory cell signalling [5].

To illustrate the role played by oxygen depletion in the FLASH effect, a recent in vitro study compared FLASH irradiation (600 Gy/s dose rate) and conventional radiotherapy (14 Gy/min dose rate) under various oxygen concentrations [8]. This study was undertaken on prostate cancer cells, irradiated with a 10 MeV electron beam under various oxygenation conditions, with the relative partial oxygen pressure ranging between 16–20%. Surviving fractions via clonogenic assays were determined after exposure to doses up to 25 Gy. The results showed no difference between the two techniques under normoxic conditions, nor under hypoxia up to 5–10 Gy radiation dose. However, above this dose range, cells irradiated with FLASH presented an increased survival, dependent on oxygen concentration, which became significant at 18 Gy. This study provides in vitro evidence supporting the oxygen dependence of FLASH effects.

A molecular dynamics simulation was performed by Abolfarth et al. [9] to study the production and interaction of reactive species around DNA for varying dose rates and oxygenation levels. In normoxic conditions at high dose rates, it was found that individual reactive oxygen species (ROS) agglomerated to form resonant or meta-stable molecular states connected by hydrogen bonds. The resulting agglomerations have a low diffusion capability and are hence non-reactive oxygen species (NROS) with limited potential for biological damage. The production of NROS was found to be reduced at lower dose rates and in hypoxic conditions resulting in a higher proportion of free ROS. It was proposed that high oxygenation levels would saturate the agglomeration process, leading ROS to again be dominant over NROS. The observed agglomeration and resulting protection of normoxic tissues at high dose rates is a potential advantage of the observed FLASH effect.

Petersson et al. [10] developed a model of oxygen depletion kinetics and the resulting oxygen enhancement ratio. It was found that the oxygen enhancement ratio was reduced for higher doses and dose-rates. The model was tested against experimental data and was able to reproduce the observed results supporting the oxygen depletion explanation of the FLASH effect.

Kusumoto et al. [11] performed an experiment to measure the yield of hydroxyl radicals for a range of dose rates using coumarin-3-carboxylic acid as a hydroxyl radical scavenger. The yield of the hydroxyl radical was found from the measured yield of 7-hydroxy-coumarin-3-carboxylic acid produced from the scavenging reactions. It was found that the hydroxyl radical yield was reduced for higher dose rates. It was proposed that the reduction in yield was the result of oxygen depletion and that the reduced yield would result in decreased indirect biological damage.

Other research suggests that FLASH therapy reduces long-term radiation effects (i.e., not the immediate cell kill), thus diminishing the side effects experienced by normal tissues post irradiation [6]. This is hypothesized to be due to reduced cell senescence, linked to the release of pro-inflammatory cytokines, with inflammatory processes remaining active in subsequent progeny for several generations. As such, decreased cell senescence indicates an overall decline in various inflammatory responses of normal tissues [6].

Jay-Gerin [12] demonstrated with Monte Carlo simulations that at the high dose rates of FLASH therapy the transient acid spikes around the path of each incident radiation particle combine to result in acidic conditions across the entire irradiated volume. It was proposed that these acidic conditions could contribute to the observed FLASH effect. Jin et al. [13] presented a computational study showing that higher dose rates reduced the proportion of circulating cells in the blood stream that were irradiated, particularly for higher doses. It was proposed that the increased sparing of circulating immune cells could contribute to the FLASH effect.

A chemical reaction kinetics model was employed by Labarbe et al. [14] to simulate the formation and decay of ROS following irradiation. It was found that the dose rate and oxygenation level had a strong effect on the lifetime of organic peroxyl radicals. At moderate oxygenation levels, higher dose rates reduced the lifetime of the organic peroxyl radicals and hence the potential biological damage. The reduction in the radical lifetime was specific for both hypoxic and high oxygenation levels. This provides a potential cause of the observed FLASH effect that does not involve oxygen depletion.

Following radiation exposure, it is the redox biology specific to normal and cancerous cells that controls the recovery from radiation damage [15]. The different redox metabolism and observed altered steady-state levels of ROS and redox metals (such as labile iron) in cancer cells, mean that normal cells can eliminate free radicals produced during irradiation more effectively [16]. Spitz et al. propose that cancer cells contain much higher levels of labile iron and transferrin receptors, resulting in magnification of Fenton reactions, catalytic processes that convert hydrogen peroxide to hydroxyl free radicals, potentially resulting in much higher oxidative damage in cancer compared to normal cells [16]. Normal cells, however, contain less labile iron and are capable of faster removal of the FLASH-induced hydroperoxides, limiting peroxidation chain reactions [16].

As a result, evidence has been put forward that the major benefit of FLASH is its reduced toxicity on normal tissues, known as the “FLASH-effect” [17]. At the same time, the literature suggests that cell-kill efficacy of FLASH is equal to conventional dose rate radiotherapy, supporting the net effect of separating the TCP and NTCP curves [17].

While some of the fundamental radiobiological processes are understood or hypothesised, much deeper understanding of FLASH-associated radiation chemistry and cellular processes is required for a safe clinical employment. Moreover, in order to implement this treatment modality scientifically, rather than phenomenologically or solely based on observations, it is important to understand the challenges imposed by FLASH to other concepts that are broadly accepted in radiation biology such as the 5 Rs [18].

## 3. Methods of FLASH Delivery and Clinical Translational Challenges

A major challenge in translating FLASH radiotherapy to the clinic is to deliver ultra-high dose rates with precision and other beam characteristics comparable to conventional radiotherapy. Currently, there are only a few devices that can deliver FLASH dose rates. Research groups have performed preclinical irradiation using electron beams from experimental linear accelerators (LINACs) [3,19] and modified clinical LINACs [20,21]. Photon beams from a LINAC may not be intense enough to reach the required high dose rates with current technology. However, X-ray beams from synchrotrons have been successfully employed [22,23]. Several authors have performed pre-clinical studies using proton beams from experimental [6] and clinical accelerators [24,25,26,27]. While pre-clinical proton FLASH therapy studies to date have used passively scattered beams, new treatment planning algorithms that also optimise the high dose rate are being developed with the potential to allow proton FLASH therapy to be delivered via pencil beam scanning techniques [28,29]. Promising novel technologies such as laser particle accelerators, Very High-Energy (>100 MeV) Electron (VHEE) beams and Pluri-directional High-energy Agile Scanning Electronic Radiotherapy (PHASER) are being explored [30,31,32,33]. Most of these devices, however, can only deliver FLASH dose rates to a limited volume and/or superficial targets. Accordingly, significant work is needed to improve and optimise the current technologies to deliver FLASH dose rates to deep-seated targets with clinically acceptable beam characteristics. Improvements are also required for treatment planning systems to enable effective FLASH radiotherapy planning [34]. Some of the typical beam delivery systems currently used for FLASH pre-clinical studies are summarised in Table 1.

Another technological challenge associated with FLASH radiotherapy beam delivery is related to the dosimetry at these extreme dose rates. Since the total dose is delivered in a very short period, accurate dosimetry is challenging, as the current protocols and equipment are designed for conventional radiotherapy where the dose rate is significantly lower. Jaccard et al. developed a reproducible passive beam monitoring system for their prototype LINAC [19]. Ionisation chambers, which are most commonly used in conventional radiotherapy, may introduce large uncertainty at these ultra-high dose rates due to saturation effects. Petersson et al. proposed a model to correct for ion recombination effects [35]. McManus et al. measured the collection efficiency of a parallel plate ionisation chamber for a high dose rate 200 MeV electron beam against a graphite colorimeter primary standard to measure correction factors to enable the potential future use of ionisation chambers for high dose rate dosimetry [36]. Jorge et al. validated TLD, alanine pellets and films for absolute dosimetry against an Advanced Markus ionization chamber [37]. Vignati et al. modelled the response of silicon dosimeters to investigate their potential for FLASH therapy dosimetry [38]. Oraiqat et al. investigated the use of ionizing radiation acoustic imaging to provide real time 3D patient dosimetry [39]. The potential of using Cerenkov light for dosimetry has also been considered [40]. Further research is required to develop monitoring chambers with clinically acceptable accuracy and reproducibility to control FLASH radiotherapy delivery.

## 4. Experimental Medicine in FLASH Radiotherapy

### 4.1. In Vitro Results with FLASH Radiotherapy

As discussed above, the radiobiological mechanisms behind the FLASH effect are not fully known and there are several hypotheses for the cellular processes that cause the advantageous normal tissue response. Looking at cell viability and DNA damage repair in vitro, Beddok et al. studied three lung cell lines (two non-transformed human lung fibroblasts MRC5 and IMR 90 and one human lung cancer) after FLASH or conventional irradiation delivered with the same LINAC (4.5 MeV electrons) [41]. Cells were exposed to 5 Gy with either FLASH (>40 Gy/s) or conventional (0.03 Gy/s) irradiation techniques. Immunofluorescence was used to assess DNA damage response and cell viability was evaluated via an MTT assay. The MTT assay showed no difference between post-irradiation results and no statistically significant differences were found between the two modalities when DNA damage was evaluated: the mean number of γH2AX foci in the cancer cell line was 30 ± 9 after conventional irradiation and 29 ± 10 after FLASH irradiation (*p* = 0.6), while for MRC5 the mean γH2AX foci was 31 ± 10 for both techniques. The fact that no differences in treatment endpoints for the normal tissue and/or tumour were found between FLASH and conventional radiotherapy, contradicts the in vivo results reported by several studies, including the current group (see Section 4.2). In view of this, it was suggested that the FLASH effect is mediated by the cellular microenvironment and/or immune response, which requires in vivo settings [41].

Proton beams delivered via laser acceleration to achieve pulsed irradiation with ultra-high dose rates were tested on HeLa cells to investigate the effect of ultra-high dose rate (10^9^ Gy/s) on cell cycle arrest, apoptotic death and colony forming ability [42]. The experiment performed at the Munich tandem accelerator facility allowed the comparison between pulsed and continuous irradiation modes. The dose delivered to a cell monolayer with the 20 MeV proton beam was 3 Gy. Immunofluorescence analyses at 10 h post-irradiation showed a significant reduction in the G_2_ fraction when pulsed radiation was used as compared to continuous irradiation, which is purportedly due to either the differences in damage complexity or to the longer duration of the G_2_ arrest after pulsed irradiation than in the continuous mode. All other studied endpoints were similar [42]. This experimental setup was later tested in mice inoculated with human tumours for in vivo assessment of pulsed and continuous proton beams versus conventionally delivered photons (see Section 4.2.2 for more details) [43]. Note that, at these ultra-high dose rates (10^9^ Gy/s), other effects might be happening, as compared to ‘conventional FLASH’, which looks at much lower dose rates (>40 Gy/s).

Another in vitro study of FLASH radiotherapy employing proton irradiation was reported by Buonanno et al. in a study undertaken on normal human lung fibroblasts [6]. Cells were exposed to therapeutic doses of 4.5 MeV proton radiation using ultra-high dose rates, up to 1000 Gy/s. For measurable endpoints, the study focused on acute and long-term normal tissue effects assessed via clonogenic survival, induction of senescence, formation of γH2AX foci and the expression of pro-inflammatory marker TGFβ. The proton dose rate employed for a FLASH effect showed no influence on acute reactions. However, long-term effects were significantly impacted by the ultra-high dose rates in terms of delayed detrimental outcome, as shown by the reduced induction of senescence and expression of pro-inflammatory markers.

A recent study that aimed to identify the mechanistic basis for the protective effect of FLASH on the normal tissue, employed a 4.5 MeV linear electron accelerator (Kinetron) to expose mouse lung (C57BL/6J wild type and Terc-/- mice) to bilateral thorax irradiation, as well as human lung fibroblast cell lines (MRC5, IMR-90) and a human lung epithelial carcinoma cell line (A-549). A clear organ sparing effect of FLASH, as compared to conventional radiotherapy was demonstrated. DNA damage response evaluation using immunofluorescence studies of γH2AX and 53BP1 foci showed minimization of DNA damage in normal lung cells in vitro, sparing of lung progenitor cells from radiation-caused damage and limitation of replicative senescence incidence by FLASH [44].

### 4.2. In Vivo Results with FLASH Radiotherapy

#### 4.2.1. Photon and Electron Beam FLASH Radiotherapy

In a study involving orthotopic lung tumours in immunocompetent mice and human lung tumour xenografts in nude mice, FLASH radiotherapy (>40 Gy/s) was delivered to assess both normal tissue complications and tumour response to high dose rates [3]. When compared to conventional protracted single dose radiotherapy (15 Gy delivered with <0.03 Gy/s), FLASH caused less lung fibrogenesis and spared normal smooth muscle and epithelial cells from apoptosis. A later study investigating the effect of dose escalation on normal tissue response showed that a 30 Gy FLASH radiotherapy was required to generate the same extent of fibrosis as 17 Gy conventional irradiation, while doses below 23 Gy FLASH induced no complications [45]. Regarding tumour control, FLASH showed comparable efficiency to conventional therapy, suggesting that ultra-high dose rate radiotherapy could eradicate tumours with fewer normal tissue complications.

A recent study by Chabi et al. [46] investigated the effect of FLASH electron therapy versus conventional therapy using a prototype electron beam LINAC (6 MeV Oriatron eRT6) on leukaemia patent derived xenografts (PDXs) and normal human haematopoiesis in NSG mice. NSG mice were conditioned with either leukaemia PDXs, human haematopoiesis or both, prior to a total body irradiation of 4 Gy. Due to the high radiosensitivity of NSG mice, the levels of leukaemia cells and normal hematopoietic were evaluated 24 h post irradiation and the cells were harvested for transplant into secondary NSG mice for long term follow-up. It was found that FLASH therapy resulted in superior killing of leukaemia cells and extended mice survival times compared to conventional therapy for two of the three PDXs examined. For the third PDX, conventional therapy resulted in greater leukaemia cell kill than FLASH therapy suggesting that inter-patient cancer variation may play a role in the effectiveness of FLASH therapy. Gene analysis was used to identify a potential genetic imprint for FLASH therapy susceptibility. It was found that FLASH therapy partially preserved hematopoietic stem/progenitor cell function, which was completely destroyed by conventional radiotherapy. The observation of FLASH sparing of healthy tissue for a lower dose of 4 Gy in a well oxygenated environment suggests that other factors beyond oxygen depletion are involved in the observed FLASH effect.

To evaluate cognitive skills in mice after whole brain irradiation with FLASH, Montay-Gruel et al. designed two separate studies assessing the efficiency of pulsed-electrons [47] and that of synchrotron generated X-ray radiation [22] to preserve normal tissue functions. In the electron study, the radiation beam was provided by a prototype electron beam LINAC (6 MeV Oriatron eRT6). The results of FLASH radiotherapy delivered with dose rates >100 Gy/s were compared with the outcome after conventional exposure (0.1 Gy/s) of a single 10 Gy dose. FLASH was shown to preserve memory and neurogenesis in the hippocampus, with over 37% of neurogenesis clusters being preserved in FLASH-irradiated mice compared to only 14% in conventionally irradiated mice [47].

In the X-ray experiment, a 10 Gy dose was also delivered as a standard for cognitive assay at a mean dose rate of 37 Gy/s (12,000 Gy/s dose rate in the slice). Conventional whole brain irradiation (0.05 Gy/s) was shown to irreversibly impair cognitive skills and induce a significant decrease in cell division in the hippocampus. X-ray FLASH therapy resulted in the preservation of memory at two and six-months post-irradiation, as well as the preservation of hippocampal cell division. The 10 Gy dose irradiation to the whole brain with various dose rates showed reduced toxicity with increased dose rate above 30 Gy/s with no additional gain above 100 Gy/s. In these two pioneering studies, the group demonstrated that neither electrons nor photons delivered at ultra-high dose rates would impact on cognitive skills after whole brain irradiation. The results suggest that the length of exposure time is a critical factor in radiation delivery [22,47].

The above results are confirmed by the work of Simmons et al. in a study that aimed to evaluate the impact of FLASH on mice brain function after whole brain irradiation using LINAC-based high energy (16–20 MeV) electron radiation [48]. Mice were exposed to 30 Gy at either FLASH dose rates (200 Gy/s for 20 MeV or 300 Gy/s for 16 MeV) or to the same dose delivered conventionally (0.13 Gy/s). The study focused on several main endpoints at 10 weeks post-irradiation: neurodegeneration, neuroinflammation and related cognitive deficits. FLASH radiotherapy was associated with reduced neuroinflammation and better preservation of cognitive functions than conventionally delivered radiation, which warrants translation into larger studies.

A recent study by Alaghband et al. [49] investigated the potential for FLASH therapy to preserve the function compared to conventional radiotherapy for the whole brain irradiation of juvenile mice. A whole brain dose of 8 Gy was delivered at a dose rate of 0.077 Gy/s for conventional radiotherapy and 4.4 × 10^6^ Gy/s for FLASH radiotherapy. It was found that FLASH therapy preserved the neurogenic niche, neurogenesis in the hippocampus and normal growth hormone levels following irradiation, while for conventional radiotherapy, all of these were degraded. FLASH therapy was also found to result in normal or near normal results in learning, memory and socialisation tests at four months post treatment, while conventional radiotherapy resulted in major deficits in these tests. This indicates that FLASH therapy has a promising potential to reduce the long-term side effects resulting from brain irradiation in the treatment of brain tumours in paediatric patients.

A compilation of the current in vivo pre-clinical studies that investigated either normal tissue complications or tumour effects after FLASH irradiation in mice, as well as in larger mammals, are presented in Table 2.

As shown above, several studies have examined FLASH effects (i.e., tissue sparing effect) on specific organs to evaluate the extent of adverse events and the potential of ultra-high dose rate radiotherapy to minimize normal tissue toxicity. Aiming to compare normal tissue effects from synchrotron and conventional radiation therapy, Smyth et al. designed a murine experiment to evaluate total and partial body irradiation outcomes from the two delivery techniques [23]. Their premise was that synchrotron radiation allows for the delivery of novel techniques, namely microbeam radiation therapy (MRT) and high dose rate synchrotron broad-beam radiotherapy (SBBR), both having the potential for elevated normal tissue sparing through a FLASH effect. The goal of the study was to determine TD_50_ values for each radiation delivery technique based on acute toxicity endpoints, by employing a dose-escalation approach (Table 2). Covering a broad range of organs owing to total body irradiation, this study is the first to report toxicity results on a larger scale by providing dose-equivalence data between conventional radiotherapy, MRT and SBBR. The study found no conclusive evidence of better normal tissue protection from SBBR delivered with a dose rate of 37 to 41 Gy/s, as compared to conventionally delivered radiation (0.05–0.06 Gy/s). In MRT (in-beam dose rate 276–319 Gy/s), the most relevant parameter influencing acute normal tissue response was the valley MRT dose. Furthermore, long-term growth impairment was observed in the mice population irradiated with MRT. More research is warranted to establish the impact of these treatment techniques and of the corresponding dose rate ranges on both acute and late normal tissue effects.

While all previously described studies were undertaken on mice, Vozenin et al. aimed to investigate the FLASH effect on larger mammals (mini pig and cat patients) for possible clinical transfer [53]. In their study, prototype LINACs, either Kinetron (4.5 MeV) or Oriatron 6e (6 MeV), were used as an electron source to deliver a wide range of dose rates [53]. One mini pig was involved in the skin-assessment study, whereby the back of the pig was exposed to doses ranging from 22 Gy to 34 Gy delivered either conventionally, (5 Gy/min) or as FLASH radiotherapy (300 Gy/s). The monitoring of skin effects took place on a weekly basis via visual examination. At 36 weeks, skin biopsies were histologically analysed. The outcome following FLASH therapy was superior to the endpoints after conventional irradiation. Minimal acute toxicities were observed with FLASH, in terms of transient depilation, but with preservation of hair follicles, while conventional dose rates lead to permanent damage of hair follicles without any regrowth after six months. Late skin toxicities were only caused by conventional treatment (skin fibro necrosis, epithelial ulceration, hyperkeratosis, inflammatory infiltration, and severe dermal remodelling), whereas FLASH caused no late adverse reactions.

The second study involved cat patients and included six cats treated for locally advanced squamous cell carcinoma of the nasal planum. This type of cancer served as a relevant model given the generally poor tumour control after conventional radiotherapy. All cats underwent a single-dose FLASH radiotherapy as part of a dose-escalation trial to identify the maximum tolerable dose. Doses to the surface of the nose were prescribed, starting from 25 Gy (based on pig skin irradiation results) to 41 Gy. After a median of 18 months follow-up time, all six cats presented with permanent depilation confined to the irradiation field, without signs of any late toxicities or damage to smelling or nutrition functions [47]. Furthermore, complete tumour response was reported for all cat patients at six months, with three cats disease free at the 18 month follow-up.

Both the pig skin results concerning normal tissue toxicity, as well as the cat trial on tumour control for locally advanced squamous cell carcinoma, demonstrated greatly promising results for various reasons: (1) the studies were designed and undertaken on larger mammals in order to allow for possible clinical transfer in human patients, (2) the results were consistent with previous outcomes demonstrated in mice, showing good reproducibility with the currently available devices and dose rates used, (3) tumour control for generally untreatable cancers with conventional radiation delivery was achieved with FLASH, (4) the maximum tolerable dose was not achieved in the cat trial, as the maximum dose used (41 Gy) showed no limiting toxicities; this result suggests the potential for dose escalation in refractory tumours.

However, not all results are positive. Contrary to the majority of FLASH study results on normal tissue toxicity, Venkatesulu et al. [54] showed that an ultra-high dose rate (35 Gy/s) exhibits no sparing effect on the immune system in cardiac and splenic models of radiation induced lymphopenia. Circulating lymphocyte levels following cardiac/splenic irradiation of mice with FLASH and conventional radiotherapy were compared, failing to show any lymphocyte sparing effect of ultra-high dose rate irradiation. Contrary to expectations, FLASH caused more severe and sustained lymphocyte depletion after both cardiac and splenic irradiation experiments. Furthermore, in a third experiment, the results of whole abdominal irradiation indicated that FLASH initiated more pronounced gastrointestinal toxicity, and led to inferior rates of mouse survival when compared to conventional radiotherapy (7 vs. 15 days, *p* = 0.0001) [54].

#### 4.2.2. Proton Beam FLASH Radiotherapy

Given the physical and radiobiological advantages of proton versus photon radiotherapy, several studies examined the potential for further differences between treatment outcome following FLASH proton radiotherapy and conventionally delivered photons.

Both pulsed and continuous proton beams (23 MeV) delivered at ultra-high dose rates were employed to investigate growth delay in the treatment of human tumour xenografts (FaDu) inoculated in mice axilla, and the outcome compared to 6 MV photon irradiation results [43]. The proton study was performed with the scanning ion microprobe SNAKE (Superconducting Nanoscope for Applied Nuclear Physics Experiments) in Munich, where an approximately 20 Gy single dose was delivered in FLASH mode (10^9^ Gy/s). The control group was exposed to LINAC-based photons with doses ranging from 10 Gy (delivered in 67 s) to 40 Gy (268 s) to allow the determination of a dose-response curve. Tumour growth delay was defined as the difference between the mean times for nonirradiated and irradiated tumours to triple their volumes. The results showed that proton doses of about 20 Gy generate tumour regression to the same extent as 30 Gy photons (see also Table 2). The radiobiological effectiveness (RBE) of pulsed protons were determined to be 1.22 ± 0.19, while for continuous proton beams, it was 1.10 ± 0.18, both being comparable to the 1.1 value for the conventional proton radiation RBE.

Conventional versus FLASH irradiation with proton beams was also tested by Girdhani et al. in order to assess possible lung sparing effects and general normal tissue outcomes in these two clinical scenarios [26]. The in vivo pre-clinical technical settings used for mice irradiation allows translation to humans, offering the possibility for further clinical research. Mice undergoing whole thorax irradiation with single-dose conventional proton therapy (1 Gy/s) of 15, 17.5 and 20 Gy were evaluated for post-therapy complications and lung fibrosis at various follow-up times, ranging from 8 to 34 weeks. The group irradiated with FLASH received ultra-high dose rates of 40 Gy/s protons and the clinical endpoints were compared to the conventionally irradiated group. Lung fibrosis was developed in 30% more mice in the conventionally irradiated group, as compared to FLASH. Furthermore, FLASH offered a superior overall survival, with a lower incidence of skin dermatitis. On a molecular level, the analysis showed differential regulation of the major DNA damage and repair pathways, as well as different immune modulation between tissues belonging to the two groups. The study concluded that FLASH proton irradiation potentially offers both acute and late normal tissue sparing due to superior immune response and DNA damage repair compared to conventionally delivered proton therapy [26].

A study by Diffenderfer et al. [27] investigated the effect of FLASH proton therapy to assess the potential for sparing normal tissue complications in the intestines compared to conventional proton therapy. Mice were treated with either whole or partial abdomen proton irradiation with a FLASH dose rate of 78 Gy/s compared to a conventional dose rate of 0.9 Gy/s. Doses of 15 Gy for whole abdomen irradiation and 12 and 18 Gy for partial abdomen irradiations were delivered. Analysis of mice that had received whole abdomen irradiation showed that mice receiving FLASH proton irradiation had a greater preservation of cells within intestinal crypts and superior regeneration of crypts 3.5 days post irradiation compared to conventionally irradiated mice. Additionally, analysis of muscle layer thickness in the intestines showed greatly reduced fibrosis for FLASH therapy compared to conventional therapy with muscle thicknesses comparable to unirradiated mice. To evaluate the efficacy of FLASH therapy in the treatment of the tumour compared to conventional proton therapy mice were injected with MH641905 pancreatic cancer cells in the flank prior to partial abdomen irradiation. Analysis of tumour growth following treatment found no difference between flash and conventional proton therapy for both 12 and 18 Gy doses.

### 4.3. Clinical Results with FLASH Radiotherapy

Clinical results are still very limited. A recent study by Bourhis et al. has reported on the first patient treated with FLASH radiotherapy [55]. The 75 year-old patient diagnosed with T-cell lymphoma underwent previous radiotherapy sessions for several cutaneous lesions showing complete response. However, due to the development of a new skin neoplasm (of 3.5 cm diameter) FLASH radiotherapy was delivered using the 5.6 MeV Oriatron designed for ultra-high dose rate treatment, to a total dose of 15 Gy in 90 ms. The aim of FLASH irradiation was to keep normal tissue toxicity to a minimum, a goal that was met with only grade 1 tissue reactions at 3 weeks post-treatment. Tumour control was also achieved, with complete response at the five-month follow-up. This first clinical report on the efficiency of FLASH to increase the therapeutic ratio warrants further evaluation and clinical applications.

## 5. Clinical Advantages of FLASH Radiotherapy Derived from Current Evidence

Among the advantages resulting from animal and human studies, the following merit highlighting:

- Greatly improved normal tissue sparing compared to the more established treatments [53,55]
- Similar tumour control to the more traditional treatment delivery techniques [56];
- Enabling dose escalation for enhanced tumour control, owing to the reduction of normal tissue complications [50].

It is also hypothesized that FLASH radiotherapy has the potential to increase the therapeutic ratio owing to differential activation of DNA damage pathways between normal and tumour cells [3] and to improve the immune response by the activation of immune pathways [26].

In view of all the above, several groups of patients might benefit from FLASH radiotherapy:

- Patients with radioresistant tumours, in need for dose escalation: glioblastomas (brain, in general), pancreas, head and neck;
- Patients with recurrent tumours in need of reirradiation with normal tissue sparing; and
- Patients with higher normal tissue radiosensitivity.

Non-conventional radiotherapeutic techniques, such as FLASH, are now in clinical focus in order to design pioneering, biologically based clinical trials [57].

## 6. Conclusions and Future Developments

Over the last years, FLASH radiotherapy has gained increasing attention due to its potential to significantly reduce normal tissue complications. Next to photons and electrons, pre-clinical studies have investigated the efficacy of FLASH delivered with proton beam radiation. While the clinical endpoints concerning normal structures are indeed promising, the radiobiological mechanisms behind ultra-high dose rate therapy are not yet fully elucidated.

Several hypotheses have been advanced to explain the distinctive response between normal and malignant cells, including differential activation of DNA damage and repair pathways, transient oxygen depletion and differences in the redox biology of oxygen metabolism. It is suggested that disparities between the decay rates of organic peroxyl radicals and organic hydroperoxides generated post-irradiation in normal versus cancer tissues, together with differences in the redox active metal ion pool (such as labile iron), are probable reasons for the distinct response [8].

The most important areas for further research to enable clinical implication of FLASH radiotherapy include:

- The evaluation of the effect of fractionated FLASH regimens [1].
- Acute normal tissue reactions seem to be diminished by the FLASH effect however, more conclusive results are needed on late toxicities and possible long-term sequelae.
- FLASH should be implemented clinically with caution in the absence of full understanding of biological mechanisms driving the radiotherapy response under FLASH conditions.

## Figures and Tables

**Table 1:** Beam delivery systems currently used for FLASH pre-clinical studies.

Experimental LINAC	Jaccard et al. (2018) [19]	Oriatron eRT6 built by PMB-Alcen	Prototype high dose-per-pulse LINAC. 6 MeV electron beam with variable dose rate (up to ~200 Gy/s at an SSD of 1 m); sometimes stated as producing a 5.6 MeV electron beam because of its softer spectrum compared to a clinical 6 MeV LINAC which uses filters and applicators. Output stability SD < 1%, but non-negligible day-to-day variations of the beam output.
Modified clinical LINACs	Schuler et al. 2016 [20]	Varian 21EX, (Varian Medical Systems, Palo Alto, CA, USA)	Average dose rates of 74 Gy/s were achieved in clinical mode at ion chamber position (9 MeV electrons). Dose rate after tuning exceeded 900 Gy/s, with technical assistance from the LINAC manufacturer. 220 Gy/s at 1-cm depth for a > 4-cm field size with 90% homogeneity throughout a 2-cm-thick volume.
	Lempart 2019 [21]	ELEKTA Precise (Elekta AB, Stockholm, Sweden)	Dose rate of >30 Gy/s and >300 Gy/s achieved at crosshair foil and wedge positions, respectively. By moving scattering foil dose rate increased to >120 Gy/s and >1000 Gy/s. 5% flatness at the crosshair position for 20 × 20 and 10 × 10 cm^2^ areas, with and without both scattering foils in the beam. 10% flatness at the wedge position.
Synchrotron	Montay-Gruel 2018 [22]	ID17 Biomedical Beamline of the ESRF (Grenoble, France).	Synchrotron X-ray, broad beam (flat beam of 50 µm). Mean energy 102 keV, mean dose rate of 37 Gy/s.
	Smyth 2018 [23]	Imaging and Medical Beamline (IMBL), Australian Synchrotron	Mean X-ray energy 124 keV, dose rate 37–41 Gy/s for SBBR (synchrotron broad-beam radiation).
Clinical accelerator	Patriarca 2018 [25]	230 MeV proton cyclotron (IBA, Belgium)	Dose rates exceeding 40 Gy/s at energies between 138 and 198 MeV were obtained. Used passive scattering setup, field size 12 × 12 mm^2^.
	Diffender et al. 2020 [27]	230 MeV proton cyclotron (IBA, Belgium)	Produced a passively scattered beam with a field size of 10 × 20 mm^2^. Dose rate increases with proton beam energy, up to over 250 Gy/s for the maximum proton energy.

**Table 2:** Normal and tumour tissue effects after FLASH irradiation in in vivo pre-clinical studies: from mice to large mammals.

Study [ref]	Organ/Tumour Evaluated	Radiation Quality and Delivery Parameters	Normal Tissue Effects/Tumour Control
FLASH vs. CRT to evaluate differential cellular response in normal and tumour tissue (Favaudon et al. 2014, 2015) [3,45]	Lung (mice)	FLASH dose rate: >40 Gy/s CRT dose rate: <0.03 Gy/s (15 Gy)	No complications with FLASH below 20 Gy at 36-weeks follow-up. Better normal tissue protection than CRT and comparable tumour control.
Electron FLASH with LINAC/Oriatron (Montay-Gruel et al. 2017, Montay-Gruel et al. 2019) [19,47,50]	Whole brain (mice)	Pulsed-electron beam FLASH to deliver 10 Gy with dose rates >100 Gy/s CRT dose rate: 0.1 Gy/s	Memory and neurogenesis preservation in the hippocampus after FLASH; Electron FLASH is superior at brain function preservation to conventional delivery. CRT led to permanent alterations in neurocognitive end points 6 months post treatment, while FLASH did not cause neuroinflammation, learning/memory deficits.
Electron FLASH study with Oriatron LINAC on juvenile mice brains (Alaghband et al. 2020) [49]	Whole brain (juvenile mice)	Whole brain dose of 8 Gy delivered at a rate of 0.077 Gy/s for the conventional treatment and 4.4 ×10^6^ Gy/s for the FLASH treatment.	FLASH therapy was found to preserve the neurogenic niche, neurogenesis in the hippocampus and normal growth hormone levels post irradiation which were all degraded by CRT. FLASH was also found to result in normal or near normal results in learning, memory and socialisation tests at 4 months post treatment, while CRT caused major deficits.
X-ray FLASH with synchrotron generated radiation (Montay-Gruel et al. 2018) [22]	Whole brain (mice)	Synchrotron X-rays: 37 Gy/s (12,000 Gy/s dose rate in the slice)	No memory deficit (preservation of spatial memory); reduced impairment of hippocampal cell division; induction of less reactive astrogliosis.
LINAC-based electron beam delivery of FLASH vs. CRT (Simmons et al. 2019) [48]	Whole brain (mice)	Single dose of 30 Gy high energy electrons (16 and 20 MeV). FLASH dose rate: 200 Gy/s for 20 MeV or 300 Gy/s for 16 MeV CRT dose rate: 0.13 Gy/s (for both energies)	FLASH showed reduced pro-inflammatory cytokines and less loss of dendritic spine density in the hippocampus, also reduced cognitive impairment and neurodegeneration compared to conventional therapy.
Electron FLASH study on treatment of Leukemia with Oriatron LINAC (Chabi et al. 2020) [46]	Total body irradiation (mice)	Whole body dose of 4 Gy delivered at a rate of <0.072 Gy/s for the conventional treatment and 200 Gy/s for the FLASH treatment	FLASH therapy was found to result in greater killing off Leukemia cells as well as longer remission delays and survival than CRT. FLASH therapy was found to preserve partial hematopoietic stem/progenitor cell function which was completely destroyed by CRT.
Proton FLASH vs. conventional 6 MV photons to evaluate tumour growth delay (Zlobinskaya et al. 2014) [43]	Hypopharyngeal squamous cell carcinoma (FaDu-inoculated mice)	FLASH dose: around 20 Gy with 10^9^ Gy/s CRT dose: 10–40 Gy delivered over 67 s to 268 s	Tumour growth delay (TGD) with photons: After 10 Gy: 12 ± 3 days After 20 Gy: 31 ± 7 days After 30 Gy: 58 ± 7 days 40 Gy resulted in complete local control at 120-day follow-up. TGD with protons: Pulsed: 34 ± 6 days Continuous: 35 ± 6 days.
Proton FLASH delivery with clinical device translatable to humans (Girdhani et al. 2019) [26]	Lung (mice)	FLASH dose rate protons: 40 Gy/s Single dose delivery of 15, 17.5 and 20 Gy Conventional proton therapy dose rate: 1 Gy/s	Targeted clinical endpoint: lung fibrosis. FLASH led to 30% reduction in lung fibrosis, lower incidence of skin dermatitis, better overall survival.
FLASH vs. CRT for total abdomen irradiation–normal tissue study (Loo et al. 2017) [51]	Whole abdomen (mice)	LINAC-based FLASH 10–22 Gy FLASH dose rate: 70–210 Gy/s CRT dose rate: 0.05 Gy/s	Survival after 20 days post irradiation with 13–19 Gy: CRT: 29% (LD50 = 14.7 Gy) FLASH: 90% (LD50 = 17.5 Gy) *p* < 0.001
Safety and efficacy of FLASH in the treatment of widespread ovarian cancer peritoneal metastases (Levy et al. 2020) [52]	Whole abdomen (mice)	LINAC-based FLASH 16 MeV with a 16 MeV scattering foil Average dose rate: 216 Gy/s at 2 Gy/pulse.	Compared to CRT, FLASH reduces early DNA damage and cell death in intestinal crypt cells, inducing higher crypt regeneration; FLASH preserves intestinal function and reduces intestinal injury caused by radiation. Tumour efficacy of FLASH was similar to CRT.
Proton FLASH delivery using modified clinical cyclotron (Diffenderfer et al. 2020) [27]	Whole abdomen/Partial abdomen and flank tumour (mice)	FLASH Proton dose rate of 78 Gy/s compared with conventional rate of 0.9 Gy/s for doses of 15 Gy for whole abdomen and 12 and 18 Gy for partial abdomen irradiations	Proton FLASH therapy increased intestinal crypt regeneration at 3.5 days post irradiation compared to conventional proton therapy as well as resulting in only minimal fibrosis. No difference in tumour volume growth post irradiation was observed between FLASH and conventional proton therapy.
Synchrotron radiation (MRT, SBBR) vs. CRT (Smyth et al. 2018) [23]	Partial body/Whole body (mice)	SBBR dose rate: 37–41 Gy/s MRT dose rate: 276–319 Gy/s (in-beam) CRT dose rate: 0.05–0.06 Gy/s	No clear evidence of improved normal tissue sparing from SBBR vs. CRT. Long-term growth impairment with MRT irradiation. TD_50_ values for TBI: 6.9 Gy (CRT), 6.7 Gy (SBBR), 120 Gy (MRT-peak), 3.8 Gy (MRT-valley). TD_50_ values for head PBI: 12.3 Gy (CRT), 13.1 Gy (SBBR), 268 Gy (MRT-peak), 7.2 Gy (MRT-valley).
Electron FLASH on large mammals to evaluate possible clinical transfer (Vozenin et al. 2019) [53]	Skin (pig) Nose skin (squamous cell carcinoma) (cats)	Kinetron/Oriatron electron radiation Pig: FLASH dose rate: 300 Gy/s CRT dose rate: 5 Gy/min Cats: All treated with a single-dose FLASH: 25–41 Gy (dose escalation trial)	Pig: Acute toxicity as transient depilation 3 weeks post-treatment with FLASH. Hair follicles preserved with FLASH and permanently destroyed with CRT. CRT induced severe late skin fibro necrosis. Cats: permanent depilation within the treated area, no late toxicities, no damage to smelling or nutrition functions. 100% complete response at 6 months; 50% disease free at 18 months post-FLASH.

**Abbreviations:** CRT = conventional radiation therapy; MRT = microbeam radiation therapy; SBBR = high dose rate synchrotron broad-beam radiotherapy; TBI = total body irradiation; PBI = partial body irradiation; LD50 = lethal dose for 50% exposed individuals.
